# Deformation Behavior of S32750 Duplex Stainless Steel Based on In Situ EBSD Technology

**DOI:** 10.3390/ma18092030

**Published:** 2025-04-29

**Authors:** Shun Bao, Han Feng, Zhigang Song, Jianguo He, Xiaohan Wu, Yang Gu

**Affiliations:** Central Iron & Steel Research Institute Co., Ltd., Beijing 100081, China; baoshun1994@163.com (S.B.); zhigangsongnercast@163.com (Z.S.); hejianguo@nercast.com (J.H.); wuxiaohan@nercast.com (X.W.); guyang@nercast.com (Y.G.)

**Keywords:** S32750 duplex stainless steel, in situ EBSD, deformation mechanism, orientation distribution

## Abstract

In this study, we investigated the two-phase hardening behavior and microstructural evolution of S32750 duplex stainless steel during the tensile deformation process. The analysis was conducted using in situ electron backscatter diffraction (EBSD), scanning electron microscopy (SEM), and microhardness testing. It was observed that strain transfer occurred between the two phases in the position away from the fracture. The ferrite phase exhibited softening, while the austenite phase underwent hardening. In the region less than 1 mm from the fracture site, both phases experienced a rapid hardening, with the maximum hardness difference between the two phases near the fracture reaching approximately 45 HV. In situ EBSD results indicate that the kernel average misorientation (KAM) value for the ferrite phase consistently exceeds that of the austenite phase during the initial stages of deformation. Conversely, in the final stages of deformation, the KAM value for austenite surpasses that of ferrite. In the initial stage of deformation, the type of grain boundaries in both phases remains largely unaltered. However, in the later stages of deformation, there is a marked increase in the number of small-angle grain boundaries within ferrite, which become approximately three times that of the large-angle grain boundaries. As deformation progresses, the maximum orientation distribution density of the ferrite phase is reduced by approximately 50%, with the preferred orientation shifting from the {100} plane to the {111} plane. In contrast, the orientation distribution of the austenite remains relatively uniform, with no significant change in the maximum orientation distribution density observed. This indicates that after substantial deformation, the rotation of ferrite grains significantly increases the deformation resistance, whereas the austenite phase continues to harden. This differential behavior leads to the continuous accumulation of strain at the phase boundaries, ultimately causing cracks to form at these boundaries and resulting in the sample’s fracture.

## 1. Introduction

The S32750 duplex stainless steel (DSS) matrix comprises two phases: ferrite and austenite. Through appropriate solution treatment, harmful phases can be eliminated and also ensure that the ferrite and austenite contents are nearly equal [1]. S32750 duplex stainless steel, renowned for its superior mechanical properties and outstanding corrosion resistance, is extensively utilized in the petrochemical and marine engineering industries [2,3,4]. After proper forging and hot rolling processes, the two phases in duplex stainless steel—ferrite and austenite—are typically uniformly distributed throughout the sheets. Due to the distinct structures of these phases, their deformation capabilities and behaviors vary significantly [5,6,7]. During the deformation process, the two phases in duplex stainless steel must not only withstand deformation together but also coordinate their responses, indicating that the underlying deformation mechanism is inherently more complex. Fu et al. [8] conducted a study on the deformation behavior of the austenitic phase in duplex stainless steel under ultra-high-cycle fatigue conditions. They found that hardening and softening phenomena are induced by the proliferation of dislocations and the plastic transfer, which in turn triggers the initiation of microcracks at the phase boundaries. The uneven distribution and transfer of load between the two phases are identified as the primary contributors to the onset of cracking. Zhang et al. [9] subjected the surface of S32750 duplex stainless steel to significant deformation using the ultrasonic surface rolling process. This treatment introduced a multitude of small-angle grain boundaries and compressive residual stress. Consequently, these alterations facilitated the enrichment of Cr_2_O_3_ during the corrosion process, thereby enhancing the steel’s overall corrosion resistance. Mariana et al. investigated the structural integrity of S32750 duplex stainless steel under thermal deformation conditions. They discovered that when the deformation exceeded 20% at 1050 °C, cracks tended to form on the side of the material. In contrast, no side cracking was observed when the deformation reached 50% at 1300 °C. This difference was primarily attributed to the formation and subsequent dissolution of the σ phase [10]. Zhao et al. [11] observed that under high-temperature conditions, a low strain rate resulted in dynamic recovery of duplex stainless steel. Conversely, at a lower temperature of 900 °C, an increase in strain rate corresponded to a higher likelihood of dynamic recrystallization occurring. During thermal deformation, temperature emerges as the predominant factor influencing the recrystallization of austenite. Notably, at high temperatures, the recrystallization behavior is largely independent of the strain rate [12]. Currently, numerous studies have been conducted on the deformation behavior of duplex stainless steel at both room temperature and high temperature. However, the relationship between the deformation behavior of the two phases and the failure behavior of the material remains underexplored.

The deformation coordination and behavior of duplex stainless steel during the deformation process play a crucial role in determining the material’s strength, plasticity, and fracture mechanism. Currently, research on the deformation behavior of duplex stainless steel predominantly concentrates on the effects of hot deformation characteristics, dynamic recovery, recrystallization, and the cold deformation process on its properties. However, there remains a significant gap in systematic and comprehensive studies regarding the two-phase hardening laws of duplex stainless steel throughout the entire deformation process, the evolution of its microstructure, and the impact of these changes on the material’s susceptibility to cracking. The interplay between the deformation behaviors of the two phases is intricately linked to the steel’s failure mechanisms. In this study, we utilized in situ EBSD technology to investigate the deformation behaviors of S32750 duplex stainless steel throughout the entire tensile process. The objective of this research is to elucidate the fracture mechanism of the steel and offer insights for enhancing its mechanical properties.

## 2. Materials and Methods

**Figure 1:**
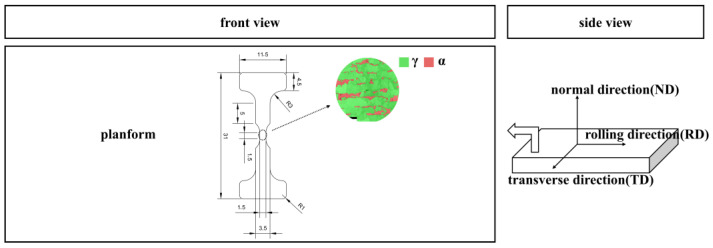
In situ tensile specimen size and sampling location.

The chemical composition of S32750 DSS hot rolled plate is as follows (in wt.%): 0.023% C, 0.31% Si, 0.67% Mn, 25.51% Cr, 6.34% Ni, 3.92% Mo, 0.25% N, and the balance Fe. To achieve a uniform two-phase structure and eliminate the precipitated phases, the test steel was subjected to solution treatment at 1080 °C, held for 40 min, and subsequently quenched in water. The mechanical property test was conducted in strict accordance with the standard GB/T 228.1-2021. The test was performed at room temperature, and the tensile rate was maintained at 0.00025 s^−1^. The testing equipment used was a GNT100 Universal Testing Machine (It is produced in Beijing, China and independently developed by Iron & Steel Research Institute Nake Testing Technology Co., Ltd.). The mechanical properties of the hot rolled plate were yield strength 570 MPa, tensile strength 816 MPa, elongation 41%, and shrinkage 68%. The in situ EBSD experiments encompassed several key components: real-time stretching using the HS4000 apparatus, scanning observation with the Thermo Scientific Apreo 2C (It is produced in the United States and held by Beijing Zhongke Kefu Technology Co., Ltd. of Beijing, China) scanning electron microscope (SEM), and acquisition of two-phase crystallology information via the EDAX Velocity Super detector. The dimensions and sampling orientation of the in situ tensile samples are illustrated in Figure 1. The applied tensile strain rate for these experiments was 2 μm/s. The microhardness test was performed using the Japanese FM-300 digital microhardness tester (It is produced by FUTURE-TECH in Japan and provided by Kunshan Fuze Testing Equipment Co., Ltd., Kunshan, China), and the FEI Quanta 650 FEG SEM (Made in the United States, provided by Beijing Yuanhaiwei Technology Co., Ltd., Beijing, China) was used to observe the fracture morphology of the sample. The grain boundaries of the two phases under different states were statistically analyzed using Aztec Crystal software (Version number: 2.1).

**Figure 2:**
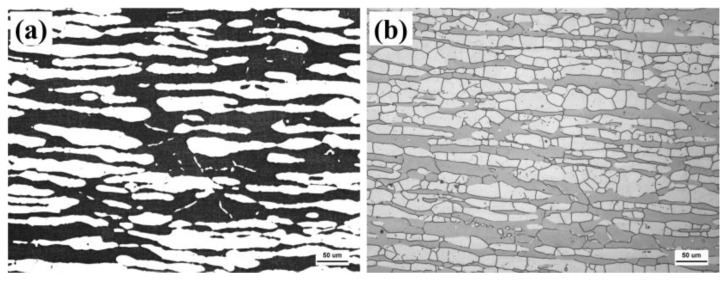
Matrix structure of S32750: morphology of the dual-phase structure (**a**); two-phase grain morphology (**b**).

The matrix of the test steel consists of ferrite and austenite. After hot rolling and solution treatment, the two phases are uniformly distributed throughout the matrix. Different etching methods reveal different morphologies. After electrolytic etching with potassium hydroxide, the two phases in the substrate are distinctly revealed, as shown in Figure 2a, where the black areas represent the ferrite phase and the white areas represent the austenite phase. The volume fractions of ferrite and austenite were 48.2% and 51.8%, respectively. After being soaked and corroded by a mixture of KOH and H_2_SO_4_, the grain boundaries of the two phases become evident, as shown in Figure 2b. The grain sizes of the two phases were calculated using Nano Measure software (version 1.2.0). The average grain sizes of ferrite and austenite were found to be 45.7 μm and 36.4 μm, respectively.

## 3. Results

### 3.1. Surface Morphology Evolution

**Figure 3:**
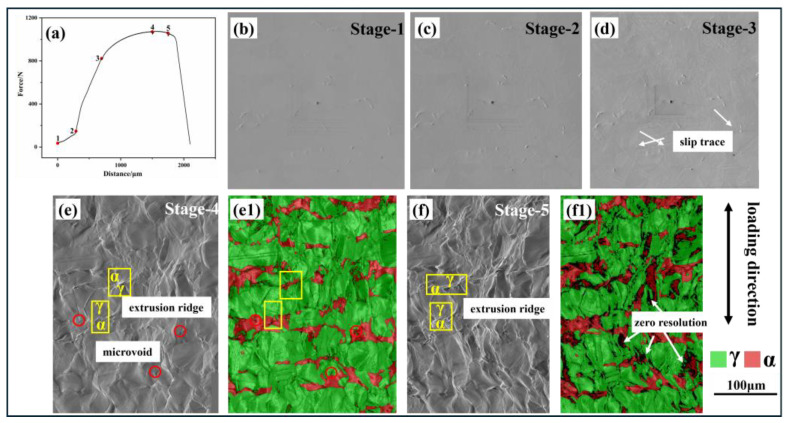
Different deformation surface morphologies of S32750 steel: (**a**) displacement-to-force curve; (**b**–**f**) represent stages 1–5; (**e1**) two-phase distribution shown in (**e**); (**f1**) two-phase distribution shown in (**f**).

Figure 3a illustrates the displacement–load curve obtained from the in situ tensile test, with points 1–5 indicating the specific stages at which in situ EBSD measurements were taken.

Figure 3b–f display the surface morphologies corresponding to these five stages. Additionally, (e1) and (f1) depict the phase distribution shown in panels (e) and (f), respectively. It was observed that during the first two stages, the slope of the displacement–load curve is relatively low, indicating that the material is undergoing elastic deformation, with no slip traces visible on the surface. The slope increases markedly in the third stage, signifying that the material has yielded and transitioned into a phase of rapid deformation. Concurrently, distinct glide paths [13] were identified on the surface of the test steel, as referenced in and depicted in Figure 3d. When a crystal is subjected to shear stress, the crystal planes are relatively displaced, leading to permanent deformation and the formation of a slip band [14] on the surface. Such slip bands are commonly observed in face-centered cubic (FCC) structural materials. On the other hand, ferrite contains a large number of screw dislocations, which are more likely to undergo cross-slip [15,16] and move on multiple slip planes. As a result, the formation of slip band structures is relatively rare.

In the fourth stage, as depicted in Figure 3e, the slip bands on the austenite surface tend to become more pronounced and deeper, leading to the formation of micropores and extrusion ridges at the phase boundaries. By the fifth stage, near the point of specimen fracture, there is a notable increase in the size of the extrusion ridges, which bear a resemblance to mountainous terrain, as illustrated in Figure 3f. The emergence and deepening of extrusion ridges and micropores are typically caused by significant plastic deformation and stress concentration within the material, indicating that fracture is imminent. Concurrently, it has been observed that significant deformation occurs at the two-phase boundary, particularly on the side adjacent to the ferrite phase, resulting in the formation of a microscopic black region, as indicated by the arrow in the Figure 3f1. The step size for the electron backscatter diffraction (EBSD) analysis was set at 0.1 μm, which means that microstructures smaller than this dimension could not be resolved with precision, hence appearing as black morphologies in the figure. As a result, duplex stainless steel is highly susceptible to failure at the phase boundaries under sustained tensile stress [17,18], as the continuous deformation can lead to the nucleation and growth of micropores, ultimately causing fracture.

### 3.2. Tensile Fracture Morphology

**Figure 4:**
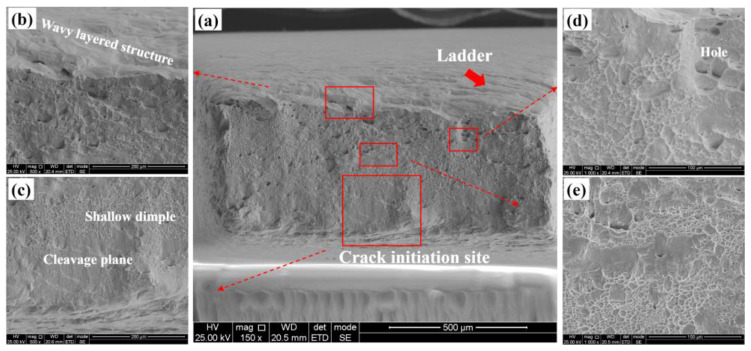
Fracture morphology of the in situ tensile specimen: (**a**) macroscopic morphology; (**b**) transient breaking zone; (**c**) crack initiation zone; (**d**) slow propagation zone; (**e**) rapid propagation zone.

Figure 4 illustrates the fracture morphology following an in situ tensile fracture. It is evident that the sample’s fracture originated from severe plastic deformation on the lower surface, which served as the primary source of cracking.

This deformation ultimately dictated the final fracture morphology. Additionally, the upper surface exhibits a ladder-like morphology, indicative of the extensive plastic deformation that occurred. Figure 4b presents an enlarged view of the upper surface area, revealing that severe plastic deformation led to the formation of a wavy, layered structure on the specimen’s surface. Figure 4c displays an enlarged view of the region proximate to the crack initiation source, characterized by a large cleavage plane and a shallow dimple. The rapid expansion rate during the early stages of crack formation results in the creation of a substantial cleavage plane. As the crack continues to propagate, its rate of expansion decelerates, leading to the progressive enlargement of the dimple morphology, as depicted in Figure 4d,e. Concurrently, the presence of some holes is also observed within this region. This is precisely how the material absorbs energy to slow the rate of crack growth.

**Figure 5:**
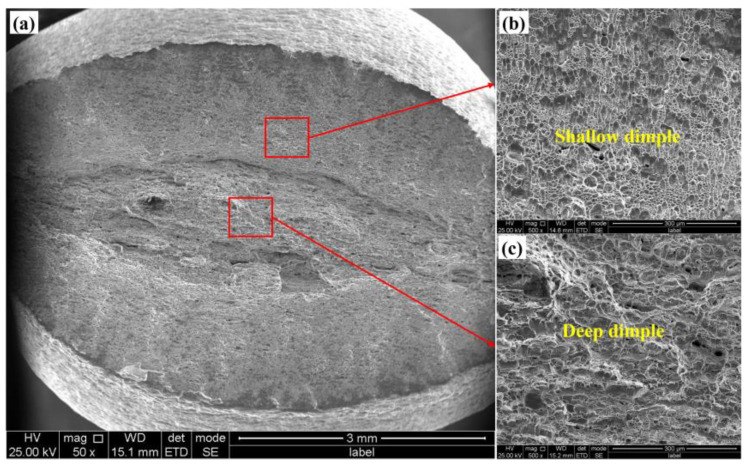
Fracture morphology of the rod tensile specimen: (**a**) macroscopic morphology; (**b**) rapid propagation zone; (**c**) slow propagation zone.

The similarities and differences in the fracture morphology between the in situ tensile plate-shaped specimens (Figure 4) and the rod-shaped specimens (Figure 5) were carefully characterized and compared.

Both sample types exhibit surface cracking and the formation of shallow dimples within the crack growth zone. However, the primary distinction lies in the crack propagation paths: plate-like tensile specimens display crack propagation from one end to the other, whereas rod-like tensile samples show cracks initiating from both ends and converging toward the middle. This results in the formation of a larger and deeper dimpled area in the rod-like samples. Additionally, it was observed that the necking in the rod-like samples consistently results in an elliptical shape. This phenomenon can be attributed to the anisotropic mechanical properties of the sample, which become evident after hot rolling. The properties of the sample in the rolling direction and perpendicular to it differ significantly, leading to greater deformation on the sides perpendicular to the rolling direction during the breaking process. Consequently, this results in the formation of an elliptical neck.

### 3.3. Variation in Two-Phase Microhardness

**Figure 6:**
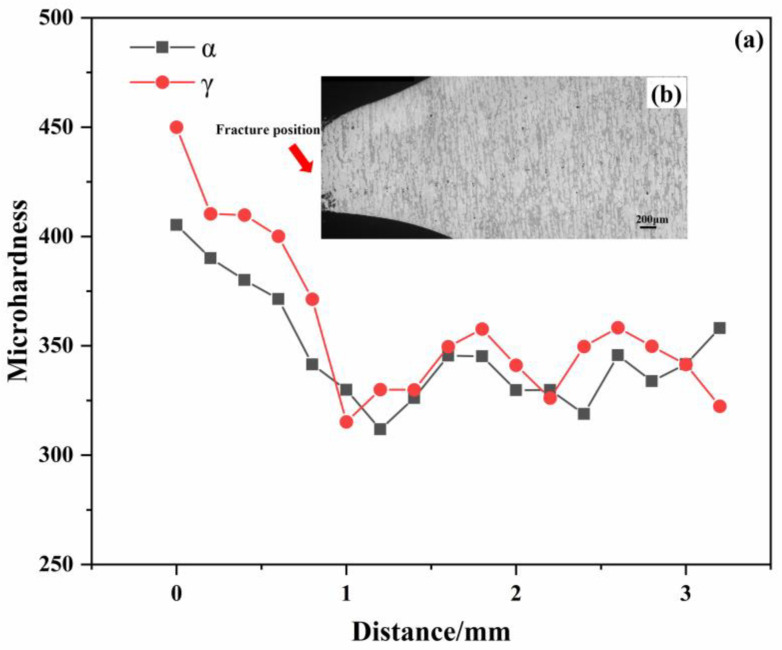
Relation between displacement and microhardness: (**a**) microhardness at different distances from the fracture surface; (**b**) the distribution of hardness marks in the matrix structure.

To analyze the strain distribution within the two phases in duplex stainless steel, microhardness measurements were conducted on the ferritic and austenitic phases at various locations. It is understood that stress varies across different positions, and consequently, the strain distribution between the two phases also differs. These measurements provide insight into the strain behavior of both phases across the entire working section. Figure 6a illustrates the relationship between the distance from the fracture and the hardness, while Figure 6b indicates the specific locations where the microhardness tests were conducted. It was observed that the hardness of the two phases does not increase in a strictly monotonic manner; instead, it exhibits a wave-like trend. Notably, the hardness of both phases increases sharply as the distance to the fracture site decreases. Approximately 3.2 mm away from the fracture surface, the microhardness of ferrite is higher than that of austenite.

However, as the distance to the fracture site decreases, it is observed that the ferrite phase begins to soften while the austenite phase hardens. This indicates that in the early stages of deformation or when the deformation is minimal, the strain distribution between the two phases is characterized by an increasing strain in austenite and a decreasing strain in ferrite. Within the interval of approximately 1 to 3 mm from the fracture, the hardening behavior of the two phases is nearly identical, exhibiting a wave-like fluctuation. Throughout this region, the microhardness of austenite consistently exceeds that of ferrite. As the distance to the fracture decreases to less than 1 mm, there is a marked increase in the hardness of both phases, with the microhardness difference reaching its peak at the point of final fracture, amounting to approximately 45 units.

## 4. Discussion

### 4.1. Evolution of Two-Phase Microscopic Strain

**Figure 7:**
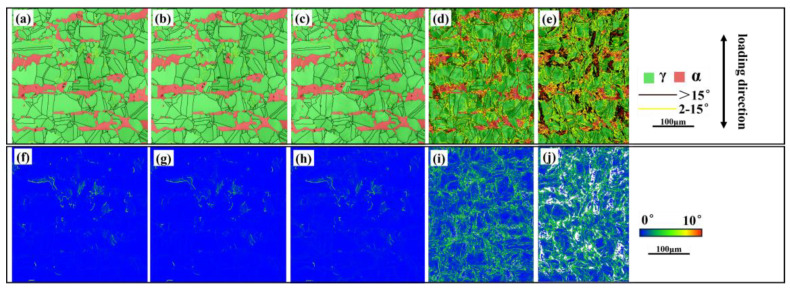
EBSD information in the five stages of the S32750 steel deformation (**a**–**e**): 1–5 grain boundary distribution; (**f**–**j**): KAM distribution.

The kernel average misorientation (KAM) is a measure that quantifies the average orientation difference in a small unit relative to its neighboring units, primarily reflecting the dislocation activity within a grain. The KAM value is positively correlated with dislocation density, making it a valuable parameter for assessing the extent of plastic strain [19]. KAM values of the two phases can serve as a measure of microscopic damage [20,21]. Higher KAM values typically indicate greater plastic deformation or higher defect density, which are often associated with microscopic damage. Figure 7a through Figure 7e depict the grain boundary distribution in the two phases across the five stages of deformation, while panels (f) through (j) illustrate the corresponding KAM value distributions. It is evident that the KAM values for both phases remain relatively stable during the first three stages. However, upon reaching the fourth stage, there is a notable increase in the KAM values, particularly at the grain and phase boundaries of the two phases. In the fifth stage, near the ferrite side of the two-phase boundary, the strain continues to intensify, leading to the formation of a smaller deformation structure, which appears as the black region in panel (e). This microstructural feature is likely a result of dislocation entanglement at the phase boundary.

**Figure 8:**
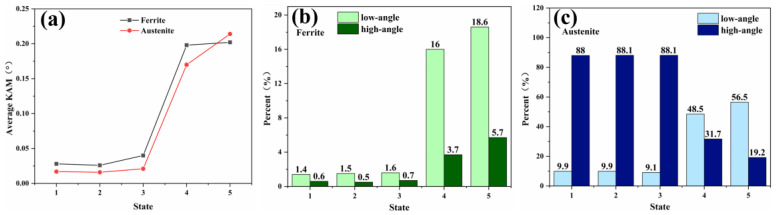
Average value of two-phase KAM and grain boundary ratio: (**a**): two-phase KAM averages for the five stages; (**b**,**c**) two-phase grain boundary ratios for the five stages.

The KAM values for the ferrite and austenite phases were calculated across the five stages of deformation, as shown in Figure 8a. Initially, in the first three stages, the average KAM values remain relatively constant, with the ferrite phase consistently showing a higher KAM value than the austenite phase. However, in the fourth and fifth stages, there is a significant increase in the average KAM values for both phases, suggesting a substantial accumulation of plastic deformation within the ferrite and austenite. By the fifth stage, the KAM value for austenite surpasses that for ferrite, indicating increased dislocation activity within the austenite phase. Concurrently, the accumulation of plastic deformation in ferrite begins to decelerate, suggesting that the ferrite reaches a state of strain saturation earlier than austenite, which retains the capacity for further deformation. As depicted in Figure 8b,c, it is observed that the proportion and growth trend of low-angle grain boundaries (LAGBs) in the two phases, ferrite and austenite, are markedly different.

During the first three stages of deformation, the number of LAGBs in both phases remains relatively constant. In the ferrite phase, the number of LAGBs exceeds that of high-angle grain boundaries (HAGBs), whereas in the austenite phase, a significant majority—approximately 90%—are HAGBs. In the final two stages of in situ tensile testing, there is a pronounced increase in the number of small-angle grain boundaries within the ferrite phase. In contrast, the austenite phase exhibits a more moderate increase in these grain boundaries. The dislocations in ferrite eventually lead to the formation of small-angle grain boundaries through the cross-entanglement of multiple slip planes, as referenced in [22]. On the other hand, the austenitic phase, characterized by lower frictional shear stress, as noted in [23], tends to accumulate dislocations at the grain and phase boundaries in the form of planar slip, resulting in a less dramatic increase in grain boundary angles.

### 4.2. Orientation Distribution of the Two Phases

**Figure 9:**
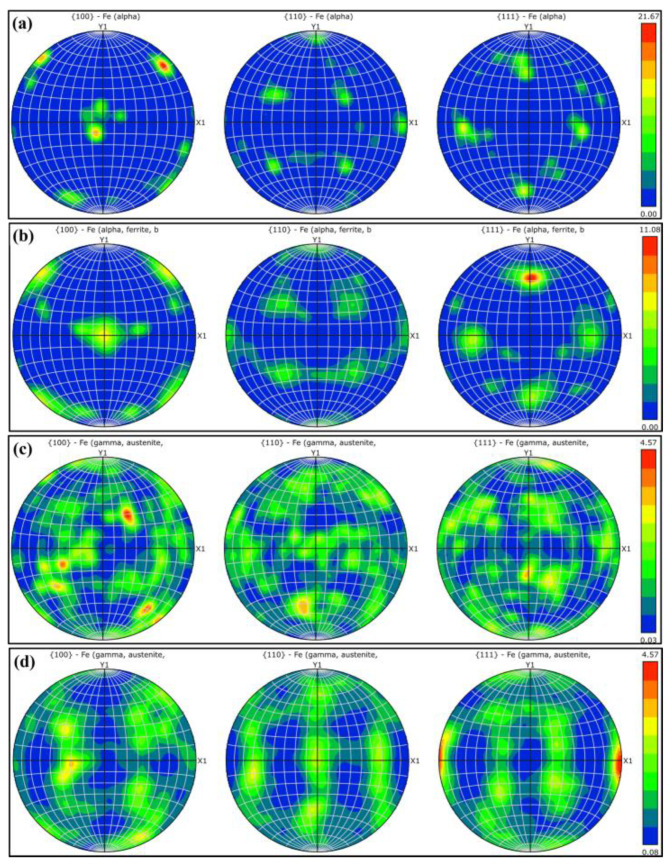
Phase orientation distribution in phases 1 and 5: ferrite (**a**,**b**); austenite (**c**,**d**).

Figure 9 illustrates the orientation distribution and intensity of the three crystallographic faces for the ferrite and austenite phases in S32750 duplex stainless steel (DSS) in the first and fifth stages of deformation.

Figure 9a,b specifically present the orientation data for the ferrite phase across these two stages. These demonstrate that from the onset to the culmination of deformation, the maximum value of the orientation distribution density for ferrite decreases to approximately half of its initial value, which is around 11. In contrast, as shown in Figure 9c,d, the maximum orientation distribution density of austenite remains constant at both stages, with a value of 4.57. The orientation distribution across the three austenite crystal faces remains relatively uniform, with minimal changes observed even after significant deformation. Conversely, at the conclusion of the deformation process, the grain orientation of ferrite exhibits a clear indication of rotation. In contrast, austenite does not undergo such rotational behavior despite the presence of large plastic deformation. It has also been reported that as stress increases in fatigue experiments, the texture of ferrite changes significantly, while that of austenite remains largely unchanged [24]. Consequently, the ferrite phase encounters increased resistance to deformation along the tensile direction, whereas the austenite phase persists in hardening according to its intrinsic hardening mechanism. This disparity leads to a substantial accumulation of strain on the ferrite side of the phase boundary, ultimately culminating in the specimen’s fracture.

## 5. Conclusions

In the present study, the tensile deformation behavior of S32750 duplex stainless steel was investigated using the in situ EBSD technique. Microhardness measurements were employed to assess the hardness variations across the entire region of the specimens. It was observed that the ferrite and austenite phases exhibited distinct deformation mechanisms. The principal conclusions drawn from this research are as follows:

1. Two-phase microhardness results: The microhardness measurements indicate that during deformation, the ferrite phase softens initially, followed by an increase in the hardness of the austenite phase, which eventually surpasses that of ferrite. Near the fracture, within a range of 1–3 mm, the hardness of both phases exhibits a wave-like pattern, suggesting that the deformation coordination between ferrite and austenite occurs through fluctuations in this region. As the distance to the fracture decreases to less than 1 mm, the hardness of both phases increases progressively, with the maximum hardness difference observed near the fracture.
2. The kernel average misorientation (KAM) value for ferrite is higher than that for austenite during the first four stages of deformation; however, this trend reverses in the fifth stage, with the KAM value for austenite surpassing that for ferrite. In the fourth and fifth stages, the small-angle grain boundaries in ferrite grow rapidly, while those in austenite increase at a relatively slower rate. In the later stages of deformation, ferrite undergoes deformation through the rapid formation of a large number of low-angle grain boundaries.
3. The changes in the orientation distribution density across the three crystal faces for the two phases reveal that ferrite grains exhibit a deflection during the deformation process, whereas austenite grains remain relatively stable. This differential behavior results in continuous straining of the austenite and the accumulation of deformation on the ferrite side of the two-phase boundary. Consequently, this localized deformation leads to the eventual fracture of the material.

## Data Availability

The original contributions presented in this study are included in the article. Further inquiries can be directed to the corresponding author.
